# 2-[(1,3-Benzo­thia­zol-2-yl)imino­meth­yl]-6-meth­oxy­phenol: a new monoclinic polymorph

**DOI:** 10.1107/S1600536813019387

**Published:** 2013-07-20

**Authors:** Md. Abu Affan, Philip G. Jessop, Md. Abdus Salam, Siti Nadiah Binti Abdul Halim, Edward R. T. Tiekink

**Affiliations:** a Department of Chemistry, Queen’s University, 90 Bader Lane, Kingston, Ontario, K7L 3N6, Canada; b Faculty of Resource Science and Technology, Universiti Malaysia Sarawak, 94300 Kota Samaharan, Sawarak, Malaysia; c Department of Chemistry, University of Malaya, 50603 Kuala Lumpur, Malaysia

## Abstract

The title compound, C_15_H_12_N_2_O_2_S, is a *P*2_1_/*c* polymorph of a previously reported *P*2_1_/*n* polymorph [Büyükgüngör *et al.* (2004 ▶). *Acta Cryst*. E**60**, o1414–o1416]. The dihedral angle between the benzo­thia­zole (r.m.s. deviation = 0.010 Å) and the benzene ring of 7.86 (6)° compares with 10.76 (10)° in the literature structure. The meth­oxy substituent is almost coplanar with the benzene ring to which it is attached [C—O—C—C torsion angle = 178.31 (14)°] and the conformation about the imine bond [1.287 (2) Å] is *E*. There is an intra­molecular O—H⋯N hydrogen bond and the hy­droxy O and thio­ether S atoms are *syn*. In the crystal, columns are formed along the *b* axis as centrosymmetric dimeric aggregates, mediated by C—H⋯O inter­actions and linked by π–π inter­actions between the thia­zole and benzene rings [centroid-to-centroid distance = 3.8256 (10) Å].

## Related literature

For background to the biological activity of organotin compounds with N-, O- and S-atom donors, see: Affan *et al.* (2009 ▶). For the structure of the *P*2_1_/*n* polymorph, see: Büyükgüngör *et al.* (2004 ▶).
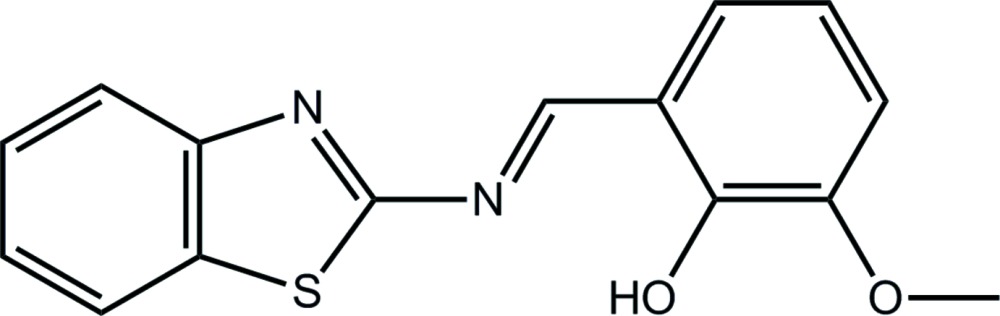


## Experimental

### 

#### Crystal data

C_15_H_12_N_2_O_2_S
*M* *_r_* = 284.33
Monoclinic, 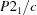

*a* = 11.6697 (11) Å
*b* = 6.0250 (6) Å
*c* = 18.6441 (18) Å
β = 94.346 (1)°
*V* = 1307.1 (2) Å^3^
*Z* = 4
Mo *K*α radiation
μ = 0.25 mm^−1^
*T* = 100 K
0.20 × 0.16 × 0.15 mm

#### Data collection

Bruker SMART APEX diffractometer
Absorption correction: multi-scan (*SADABS*; Sheldrick, 1996 ▶) *T*_min_ = 0.669, *T*_max_ = 0.746
15750 measured reflections
2983 independent reflections
2404 reflections with *I* > 2σ(*I*)
*R*_int_ = 0.046

#### Refinement

*R*[*F* ^2^ > 2σ(*F* ^2^)] = 0.036
*wR*(*F* ^2^) = 0.095
*S* = 1.05
2983 reflections
183 parameters
H-atom parameters constrained
Δρ_max_ = 0.24 e Å^−3^
Δρ_min_ = −0.26 e Å^−3^

### 

Data collection: *SMART* (Bruker, 2009 ▶); cell refinement: *SAINT* (Bruker, 2009 ▶); data reduction: *SAINT*; program(s) used to solve structure: *SHELXS97* (Sheldrick, 2008 ▶); program(s) used to refine structure: *SHELXL97* (Sheldrick, 2008 ▶); molecular graphics: *ORTEP-3 for Windows* (Farrugia, 2012 ▶), *QMol* (Gans & Shalloway, 2001 ▶) and *DIAMOND* (Brandenburg, 2006 ▶); software used to prepare material for publication: *publCIF* (Westrip, 2010 ▶).

## Supplementary Material

Crystal structure: contains datablock(s) global, I. DOI: 10.1107/S1600536813019387/su2623sup1.cif

Structure factors: contains datablock(s) I. DOI: 10.1107/S1600536813019387/su2623Isup2.hkl

Supplementary material file. DOI: 10.1107/S1600536813019387/su2623Isup3.cml

Additional supplementary materials:  crystallographic information; 3D view; checkCIF report

## Figures and Tables

**Table 1:** Hydrogen-bond geometry (Å, °)

*D*—H⋯*A*	*D*—H	H⋯*A*	*D*⋯*A*	*D*—H⋯*A*
O1—H1O⋯N2	0.84	1.88	2.6167 (17)	146
C6—H6⋯O2^i^	0.95	2.56	3.424 (2)	151

Symmetry code: (i) 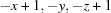.

**Fig. 1.**
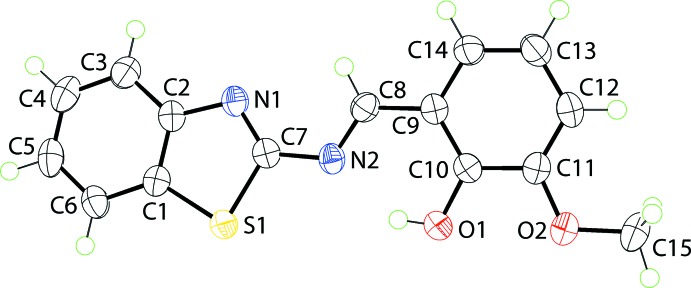
The molecular structure of (I), showing the atom-labelling scheme and displacement ellipsoids at the 70% probability level.

**Fig. 2.**
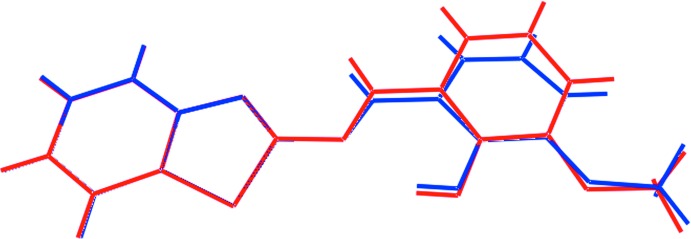
Superimposition of the molecule in (I) (red image) on that found in the polymorph (blue image). The five-membered rings have been superimposed.

**Fig. 3.**
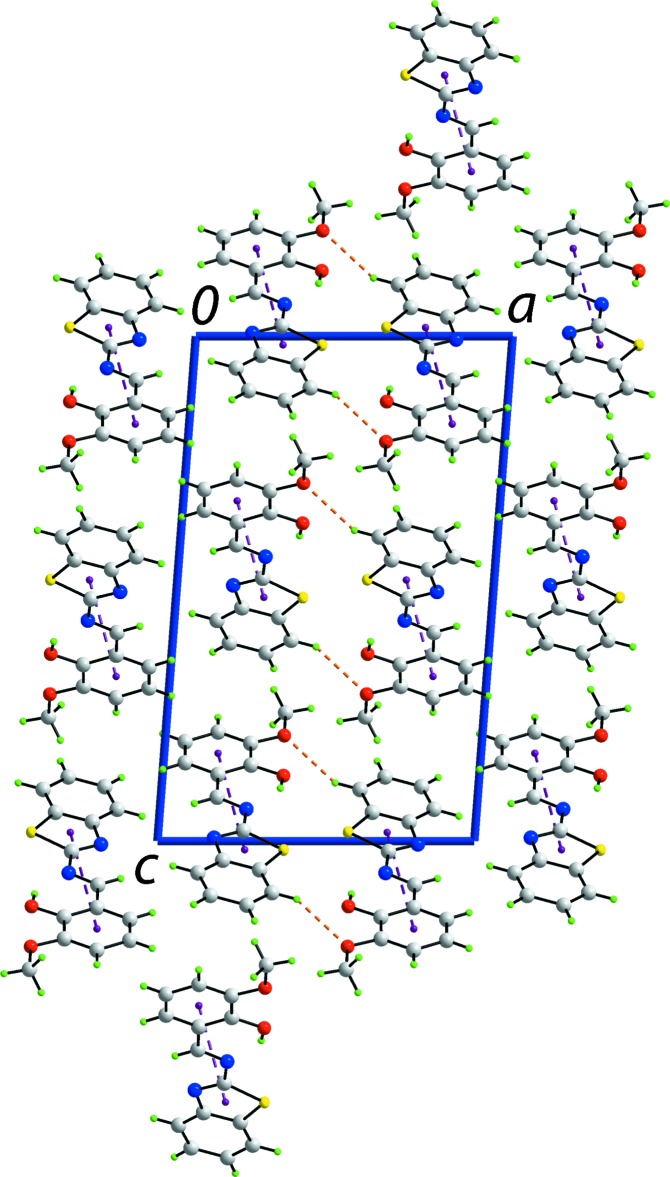
A view in projection down the b axis of the unit-cell contents for (I). The C—H···O and π—π interactions are shown as orange and purple dashed lines, respectively.

## supplementary crystallographic information

### Comment

The title compound, (I), was prepared in connection with on-going studies of organotin compounds with *N*, *O* and *S* donors for evaluation for biological activity (Affan *et al.*, 2009).

In (I), Fig. 1, the dihedral angle between the benzothiazole (r.m.s. deviation = 0.010 Å) and the benzene ring is 7.86 (6)°. This, coupled with the observation that the methoxy substituent is coplanar with the benzene ring to which it is attached, the C15—O2—C11—C10 torsion angle is 178.31 (14)°, indicates that the molecule is approximately planar. Indeed, the r.m.s. deviation for all 20 non-hydrogen atoms is 0.083 Å, with maximum deviations of 0.123 (1) Å for the S1 atom and -0.148 (2) Å for C3. As seen from the overlay diagram in Fig. 2, this conformation is similar to that found in the *P*2_1_/*n* polymorph, for which the dihedral angle between the benzothiazole and benzene ring is 10.76 (10)° (Büyükgüngör *et al.*, 2004). The coplanarity about the imine C8═N2 bond [1.287 (2) Å], with an *E* conformation, enables the formation of an intramolecular O—H···N hydrogen bond, Table 1. The hydroxyl-O and thioether-S atoms are *syn*.

In the crystal packing, centrosymmetrically related molecules associate into dimers *via* C—H···O interactions and stack in columns along the *b* axis *via*π–π interactions between the thiazole and benzene rings [inter-centroid distance = 3.8256 (10) Å, angle of inclination = 7.47 (8)° for symmetry operation *x*, -1 + *y*, *z*], Fig. 3 and Table 1.

### Experimental

2-Aminobenzothiazole (0.765 g, 5 mmol) in ethanol (10 ml) was added to an ethanolic solution of 4-(aminomethyl)-2-methoxyphenol (0.751 g, 5 mmol) and the reaction mixture was refluxed for 2 h. After cooling, a yellow solid was filtered off and washed with cold ethanol. The title compound (I) was obtained after recrystallization from its methanol solution [m.p. 466–468 K, yield 1.18 g (78%)].

### Refinement

Carbon-bound H atoms were placed in calculated positions [C—H = 0.95 to 0.98 Å, *U*_iso_(H) = 1.5*U*_eq_(C-methyl) and 1.2U_eq_(C) for other H atoms] and were included in the refinement in the riding-model approximation. The hydroxyl H atom was treated similarly [O—H = 0.84 Å; *U*_iso_(H) = 1.5*U*_eq_(O)].

### Crystal data

**Table:**

C_15_H_12_N_2_O_2_S	*F*(000) = 592
*M**_r_* = 284.33	*D*_x_ = 1.445 Mg m^−^^3^
Monoclinic, *P*2_1_/*c*	Mo *K*α radiation, λ = 0.71073 Å
Hall symbol: -P 2ybc	Cell parameters from 2808 reflections
*a* = 11.6697 (11) Å	θ = 2.7–26.6°
*b* = 6.0250 (6) Å	µ = 0.25 mm^−^^1^
*c* = 18.6441 (18) Å	*T* = 100 K
β = 94.346 (1)°	Block, yellow
*V* = 1307.1 (2) Å^3^	0.20 × 0.16 × 0.15 mm
*Z* = 4	

### Data collection

**Table:**

Bruker SMART APEX diffractometer	2983 independent reflections
Radiation source: fine-focus sealed tube	2404 reflections with *I* > 2σ(*I*)
Graphite monochromator	*R*_int_ = 0.046
φ and ω scans	θ_max_ = 27.5°, θ_min_ = 2.2°
Absorption correction: multi-scan (*SADABS*; Sheldrick, 1996)	*h* = −15→15
*T*_min_ = 0.669, *T*_max_ = 0.746	*k* = −7→7
15750 measured reflections	*l* = −23→24

### Refinement

**Table:**

Refinement on *F*^2^	Primary atom site location: structure-invariant direct methods
Least-squares matrix: full	Secondary atom site location: difference Fourier map
*R*[*F*^2^ > 2σ(*F*^2^)] = 0.036	Hydrogen site location: inferred from neighbouring sites
*wR*(*F*^2^) = 0.095	H-atom parameters constrained
*S* = 1.05	*w* = 1/[σ^2^(*F*_o_^2^) + (0.0424*P*)^2^ + 0.3573*P*] where *P* = (*F*_o_^2^ + 2*F*_c_^2^)/3
2983 reflections	(Δ/σ)_max_ = 0.001
183 parameters	Δρ_max_ = 0.24 e Å^−^^3^
0 restraints	Δρ_min_ = −0.26 e Å^−^^3^

### Special details

**Table:**

Geometry. All s.u.'s (except the s.u. in the dihedral angle between two l.s. planes) are estimated using the full covariance matrix. The cell s.u.'s are taken into account individually in the estimation of s.u.'s in distances, angles and torsion angles; correlations between s.u.'s in cell parameters are only used when they are defined by crystal symmetry. An approximate (isotropic) treatment of cell s.u.'s is used for estimating s.u.'s involving l.s. planes.
Refinement. Refinement of *F*^2^ against ALL reflections. The weighted *R*-factor *wR* and goodness of fit *S* are based on *F*^2^, conventional *R*-factors *R* are based on *F*, with *F* set to zero for negative *F*^2^. The threshold expression of *F*^2^ > 2σ(*F*^2^) is used only for calculating *R*-factors(gt) *etc*. and is not relevant to the choice of reflections for refinement. *R*-factors based on *F*^2^ are statistically about twice as large as those based on *F*, and *R*-factors based on ALL data will be even larger.

### Fractional atomic coordinates and isotropic or equivalent isotropic displacement parameters (Å^2^)

**Table:**

	*x*	*y*	*z*	*U*_iso_*/*U*_eq_	
S1	0.40036 (3)	−0.28845 (7)	0.51769 (2)	0.02579 (13)	
O1	0.38182 (9)	0.30422 (19)	0.37054 (6)	0.0291 (3)	
H1O	0.3754	0.1918	0.3966	0.044*	
O2	0.37477 (10)	0.6591 (2)	0.29096 (6)	0.0319 (3)	
N1	0.17783 (11)	−0.3057 (2)	0.49353 (7)	0.0242 (3)	
N2	0.27272 (11)	−0.0044 (2)	0.43688 (7)	0.0233 (3)	
C1	0.32893 (13)	−0.5026 (3)	0.55699 (8)	0.0231 (3)	
C2	0.21017 (13)	−0.4851 (3)	0.53783 (8)	0.0232 (3)	
C3	0.13496 (15)	−0.6425 (3)	0.56295 (9)	0.0301 (4)	
H3	0.0549	−0.6355	0.5495	0.036*	
C4	0.17954 (16)	−0.8081 (3)	0.60766 (10)	0.0335 (4)	
H4	0.1292	−0.9150	0.6257	0.040*	
C5	0.29730 (16)	−0.8223 (3)	0.62712 (9)	0.0318 (4)	
H5	0.3253	−0.9383	0.6582	0.038*	
C6	0.37344 (15)	−0.6718 (3)	0.60217 (9)	0.0278 (4)	
H6	0.4535	−0.6826	0.6152	0.033*	
C7	0.26852 (13)	−0.1942 (3)	0.47956 (8)	0.0228 (3)	
C8	0.17759 (14)	0.0866 (3)	0.41305 (8)	0.0241 (3)	
H8	0.1071	0.0233	0.4253	0.029*	
C9	0.17491 (13)	0.2822 (3)	0.36818 (8)	0.0225 (3)	
C10	0.27665 (13)	0.3830 (3)	0.34943 (8)	0.0221 (3)	
C11	0.27064 (14)	0.5760 (3)	0.30645 (8)	0.0235 (3)	
C12	0.16509 (14)	0.6647 (3)	0.28398 (8)	0.0265 (4)	
H12	0.1612	0.7948	0.2551	0.032*	
C13	0.06360 (15)	0.5648 (3)	0.30329 (9)	0.0298 (4)	
H13	−0.0088	0.6272	0.2876	0.036*	
C14	0.06870 (14)	0.3774 (3)	0.34480 (9)	0.0274 (4)	
H14	−0.0004	0.3108	0.3580	0.033*	
C15	0.37379 (17)	0.8514 (3)	0.24585 (10)	0.0353 (4)	
H15A	0.3302	0.8193	0.2000	0.053*	
H15B	0.4529	0.8914	0.2369	0.053*	
H15C	0.3377	0.9751	0.2697	0.053*	

### Atomic displacement parameters (Å^2^)

**Table:**

	*U*^11^	*U*^22^	*U*^33^	*U*^12^	*U*^13^	*U*^23^
S1	0.0245 (2)	0.0231 (2)	0.0297 (2)	0.00138 (16)	0.00111 (16)	0.00382 (17)
O1	0.0241 (6)	0.0283 (7)	0.0344 (7)	0.0017 (5)	−0.0005 (5)	0.0084 (5)
O2	0.0308 (6)	0.0303 (7)	0.0348 (7)	−0.0024 (5)	0.0041 (5)	0.0105 (5)
N1	0.0272 (7)	0.0211 (7)	0.0243 (7)	0.0006 (5)	0.0013 (5)	0.0008 (5)
N2	0.0280 (7)	0.0181 (7)	0.0234 (7)	0.0004 (5)	0.0006 (5)	0.0010 (5)
C1	0.0289 (8)	0.0202 (8)	0.0208 (7)	0.0005 (6)	0.0047 (6)	−0.0015 (6)
C2	0.0293 (8)	0.0198 (8)	0.0206 (7)	0.0020 (6)	0.0036 (6)	−0.0013 (6)
C3	0.0318 (9)	0.0286 (9)	0.0307 (9)	−0.0012 (7)	0.0065 (7)	0.0018 (7)
C4	0.0412 (10)	0.0267 (9)	0.0342 (9)	−0.0031 (8)	0.0122 (8)	0.0049 (7)
C5	0.0466 (11)	0.0239 (9)	0.0257 (9)	0.0065 (8)	0.0075 (8)	0.0051 (7)
C6	0.0327 (9)	0.0264 (9)	0.0244 (8)	0.0060 (7)	0.0027 (7)	0.0016 (7)
C7	0.0263 (8)	0.0210 (8)	0.0209 (8)	0.0015 (6)	0.0008 (6)	−0.0021 (6)
C8	0.0259 (8)	0.0223 (8)	0.0242 (8)	−0.0028 (6)	0.0018 (6)	−0.0014 (6)
C9	0.0272 (8)	0.0201 (8)	0.0199 (7)	0.0001 (6)	0.0000 (6)	−0.0018 (6)
C10	0.0251 (8)	0.0212 (8)	0.0197 (7)	0.0016 (6)	−0.0006 (6)	−0.0028 (6)
C11	0.0293 (8)	0.0209 (8)	0.0203 (7)	−0.0023 (6)	0.0019 (6)	−0.0015 (6)
C12	0.0345 (9)	0.0226 (8)	0.0218 (8)	0.0028 (7)	−0.0014 (7)	0.0024 (6)
C13	0.0288 (9)	0.0290 (9)	0.0310 (9)	0.0046 (7)	−0.0028 (7)	0.0028 (7)
C14	0.0254 (8)	0.0260 (9)	0.0305 (9)	−0.0013 (7)	0.0003 (7)	0.0009 (7)
C15	0.0453 (11)	0.0259 (9)	0.0356 (10)	−0.0051 (8)	0.0092 (8)	0.0063 (8)

### Geometric parameters (Å, º)

**Table:**

S1—C1	1.7289 (16)	C5—C6	1.375 (2)
S1—C7	1.7404 (16)	C5—H5	0.9500
O1—C10	1.3466 (18)	C6—H6	0.9500
O1—H1O	0.8400	C8—C9	1.444 (2)
O2—C11	1.3653 (19)	C8—H8	0.9500
O2—C15	1.431 (2)	C9—C10	1.401 (2)
N1—C7	1.296 (2)	C9—C14	1.405 (2)
N1—C2	1.395 (2)	C10—C11	1.411 (2)
N2—C8	1.287 (2)	C11—C12	1.378 (2)
N2—C7	1.396 (2)	C12—C13	1.400 (2)
C1—C6	1.397 (2)	C12—H12	0.9500
C1—C2	1.409 (2)	C13—C14	1.368 (2)
C2—C3	1.397 (2)	C13—H13	0.9500
C3—C4	1.377 (2)	C14—H14	0.9500
C3—H3	0.9500	C15—H15A	0.9800
C4—C5	1.397 (3)	C15—H15B	0.9800
C4—H4	0.9500	C15—H15C	0.9800
			
C1—S1—C7	88.67 (8)	N2—C8—H8	119.1
C10—O1—H1O	109.5	C9—C8—H8	119.1
C11—O2—C15	116.98 (13)	C10—C9—C14	119.33 (14)
C7—N1—C2	109.40 (13)	C10—C9—C8	121.11 (14)
C8—N2—C7	118.64 (14)	C14—C9—C8	119.53 (15)
C6—C1—C2	121.41 (15)	O1—C10—C9	123.02 (14)
C6—C1—S1	129.12 (13)	O1—C10—C11	117.49 (14)
C2—C1—S1	109.47 (12)	C9—C10—C11	119.50 (14)
C3—C2—N1	125.17 (15)	O2—C11—C12	125.64 (15)
C3—C2—C1	119.68 (15)	O2—C11—C10	114.56 (14)
N1—C2—C1	115.15 (14)	C12—C11—C10	119.80 (15)
C4—C3—C2	118.49 (16)	C11—C12—C13	120.61 (15)
C4—C3—H3	120.8	C11—C12—H12	119.7
C2—C3—H3	120.8	C13—C12—H12	119.7
C3—C4—C5	121.37 (16)	C14—C13—C12	119.95 (15)
C3—C4—H4	119.3	C14—C13—H13	120.0
C5—C4—H4	119.3	C12—C13—H13	120.0
C6—C5—C4	121.34 (16)	C13—C14—C9	120.81 (16)
C6—C5—H5	119.3	C13—C14—H14	119.6
C4—C5—H5	119.3	C9—C14—H14	119.6
C5—C6—C1	117.70 (16)	O2—C15—H15A	109.5
C5—C6—H6	121.1	O2—C15—H15B	109.5
C1—C6—H6	121.1	H15A—C15—H15B	109.5
N1—C7—N2	127.11 (14)	O2—C15—H15C	109.5
N1—C7—S1	117.30 (12)	H15A—C15—H15C	109.5
N2—C7—S1	115.58 (11)	H15B—C15—H15C	109.5
N2—C8—C9	121.88 (15)		
			
C7—S1—C1—C6	−179.01 (16)	C1—S1—C7—N2	−179.79 (12)
C7—S1—C1—C2	0.03 (12)	C7—N2—C8—C9	−179.54 (13)
C7—N1—C2—C3	−179.18 (15)	N2—C8—C9—C10	−0.8 (2)
C7—N1—C2—C1	0.46 (19)	N2—C8—C9—C14	−178.75 (15)
C6—C1—C2—C3	−1.5 (2)	C14—C9—C10—O1	179.00 (14)
S1—C1—C2—C3	179.37 (12)	C8—C9—C10—O1	1.1 (2)
C6—C1—C2—N1	178.85 (14)	C14—C9—C10—C11	−1.1 (2)
S1—C1—C2—N1	−0.28 (17)	C8—C9—C10—C11	−178.97 (14)
N1—C2—C3—C4	−178.67 (15)	C15—O2—C11—C12	−1.8 (2)
C1—C2—C3—C4	1.7 (2)	C15—O2—C11—C10	178.31 (14)
C2—C3—C4—C5	−0.9 (3)	O1—C10—C11—O2	0.5 (2)
C3—C4—C5—C6	−0.2 (3)	C9—C10—C11—O2	−179.46 (13)
C4—C5—C6—C1	0.5 (2)	O1—C10—C11—C12	−179.39 (14)
C2—C1—C6—C5	0.4 (2)	C9—C10—C11—C12	0.7 (2)
S1—C1—C6—C5	179.32 (13)	O2—C11—C12—C13	−179.97 (15)
C2—N1—C7—N2	179.60 (14)	C10—C11—C12—C13	−0.1 (2)
C2—N1—C7—S1	−0.44 (17)	C11—C12—C13—C14	0.0 (3)
C8—N2—C7—N1	6.8 (2)	C12—C13—C14—C9	−0.4 (3)
C8—N2—C7—S1	−173.12 (12)	C10—C9—C14—C13	0.9 (2)
C1—S1—C7—N1	0.25 (13)	C8—C9—C14—C13	178.87 (15)

### Hydrogen-bond geometry (Å, º)

**Table:**

*D*—H···*A*	*D*—H	H···*A*	*D*···*A*	*D*—H···*A*
O1—H1*O*···N2	0.84	1.88	2.6167 (17)	146
C6—H6···O2^i^	0.95	2.56	3.424 (2)	151

Symmetry code: (i) −*x*+1, −*y*, −*z*+1.
